# The recovery of added nematode eggs from horse and sheep faeces by three methods

**DOI:** 10.1186/s12917-017-1326-7

**Published:** 2018-01-05

**Authors:** Antonio Bosco, Maria Paola Maurelli, Davide Ianniello, Maria Elena Morgoglione, Alessandra Amadesi, Gerald C. Coles, Giuseppe Cringoli, Laura Rinaldi

**Affiliations:** 1 Department of Veterinary Medicine and Animal Production, University of Naples Federico II, CREMOPAR Campania Region, Naples, Italy; 2 University of Bristol, School of Veterinary Sciences, Langford House, Bristol, BS40 5DU UK

**Keywords:** Mini-FLOTAC, Fill-FLOTAC, Nematodes, Horses, Sheep

## Abstract

**Background:**

Nematode infections in horses are widespread across the world. Increasing levels of anthelmintic resistance, reported worldwide in equine parasites, have led to the creation of programs for the control of nematodes based on faecal egg counts (FEC). To improve nematode egg counting in equine faecal samples and establish whether the matrix of equine faeces or the eggs affect the counts, the analytical sensitivity, accuracy and precision of Mini-FLOTAC (combined with Fill-FLOTAC), McMaster and Cornell-Wisconsin techniques were compared. Known numbers of eggs extracted from equine or ovine faeces were added to egg free ovine and equine faeces to give counts of 10, 50, 200 and 500 eggs per gram (EPG) of faeces.

**Results:**

The Cornell-Wisconsin significantly underestimated egg counts and McMaster showed a low analytical sensitivity, revealing 100% of sensitivity only for concentrations greater than 200 EPG. EPG values detected by Mini-FLOTAC did not differ significantly from expected counts at any level of egg density.

**Conclusions:**

Mini-FLOTAC combined to Fill-FLOTAC which provides an accurate method of weighing without need for a balance and filtering out debris, could be used for FEC on the farm as well as in the laboratory.

**Electronic supplementary material:**

The online version of this article (10.1186/s12917-017-1326-7) contains supplementary material, which is available to authorized users.

## Background

Nematodes which infect horses are clinically important across the world and anthelmintic resistance (AR) is becoming increasingly prevalent [1]. The problem of AR has led to the creation of programs for the control of nematodes based on faecal egg counts (FEC). More accurate and precise FEC methods need to be included in studies evaluating any parasite control program, emphasizing the requirement for simple, reliable and sensitive diagnostic tools and preferably suitable to assess both the intensity of infections and the efficacy of drugs on horse farms [1]. Sources of potential error include the method of sampling, flotation solution used, sample dilution, counting procedures [2–4], faecal moisture [5], and the storage or preservation of faeces [3, 6]. In order to evaluate which FEC technique is characterized by higher analytical sensitivity (the smallest number of parasitic elements in a sample that can be detected accurately by a given technique), accuracy (how well the observed value agrees with the ‘true’ value) and precision (how well repeated observations agree with one another), eggs extracted from equine and ovine faecal samples and added to egg free samples were counted by three FEC techniques: Mini-FLOTAC, modified McMaster and Cornell-Wisconsin.

## Methods

Faecal samples with positive and negative FEC were collected from adult sheep and horses stabled in paddock of farms located in southern Italy. Each sample was analyzed 5 times by the FLOTAC basic technique [7] with an analytical sensitivity of 1 egg per gram (EPG) of faeces to determine the presence/absence of nematode eggs, i.e. cyathostomes for horses and gastrointestinal nematodes (*Trichostrongylus*, *Haemonchus* and *Teladorsagia*) for sheep. Nematode eggs were extracted from the positive samples using the mass recovery method, i.e., a method that employs 4 sieves of different dimension (1 mm, 250 μm, 212 μm and 38 μm) in order to separate the eggs from the faeces. Then ten aliquots of 0.1 ml each were taken and the number of eggs counted [8]. A series of cross-contaminations were performed: nematode extracted from horses’ faeces were used to contaminate negative horse and sheep faeces and *vice versa*. The egg suspensions were added to the negative faeces (250 g) and thoroughly homogenized to give four faecal samples (250 g each) for each EPG level (10, 50, 200 and 500).

Each sample was analyzed using satured sodium chloride solution (specific gravity = 1.200) by three FEC techniques: Mini-FLOTAC combined with Fill-FLOTAC [9–11], modified McMaster technique [12] and Cornell-Wisconsin technique [13]. After a thorough homogenization from each faecal sample for each EPG level, 60 g were weighted for Mini-FLOTAC, 36 g for McMaster chamber, 36 g for McMaster grid and 60 g for Cornell-Wisconsin. In total twelve replicates were used for each method and for each EPG level (10, 50, 200 and 500) using single faecal samples. The weight of faeces used, dilution ratio, reading volume and analytical sensitivity of each technique are shown in Table 1. Fill-FLOTAC enables the first four step of the Mini-FLOTAC technique i.e. sample collection and weighing, homogenization, filtration and filling of Mini-FLOTAC chamber [9, 11]. The repeatability of the 5 g size of Fill-FLOTAC to measure 5 g of faeces using horse and sheep samples was measured 10 times.

**Table 1:** Schematic features of Mini-FLOTAC, McMaster (grid and chamber) and Cornell-Wisconsin techniques

FEC Techniques	Amount of faeces used (grams)	Dilution Ratio	Reading Volume (ml)	Reading Area (mm^2^)	Analytical sensitivity (EPG)
Mini-FLOTAC	5	1:10	2.0	648	5
McMaster *grid*	3	1:15	0.30	200	50
McMaster *chamber*	3	1:15	1.0	648	15
Cornell-Wisconsin	5	1:10	10	324	1

The weight of faeces used for each replicate, dilution ratio, reading volume, reading area and analytical sensitivity of Mini-FLOTAC, two versions of McMaster (grid and chamber) and Cornell-Wisconsin egg counting

### Statistical analysis

A coefficient of variation [(standard deviation divided by mean count times) *100] was calculated for each set of replicate counts for each method and level of EPG. The coefficient of variation showed the precision of the method [14] that refers to the closeness of two or more measurements to each other. Mean of eggs (X) showed the accuracy of the method that describe the closeness of a measurement to the true value.

The raw counts from each sample were multiplied by the appropriate multiplication factor (5 for Mini-FLOTAC, 50 for McMaster grid, 15 for McMaster chamber and 1 for Cornell-Wisconsin), and then, the mean of the replicate counts for each sample was calculated.

The analytical sensitivity of tests across the different levels of egg excretion for each technique was evaluated using line graphs.

Boxplots (indicating median, percentiles and outliers) were used to estimate the precision and accuracy of each technique for each of the four levels of egg cross-contamination. A no parametric test, i.e. Spearman rank correlation (*rho*), was used to examine any association between true and observed egg counts. For each FEC technique at each level of egg count, the percentage recovery was calculated to assess the level of over- or under- estimation of FEC result (measurement error) using the following formula: % egg recovery = 100 - (true FEC - observed FEC) / true FEC * 100. Significance testing was set at *p* < 0.05. Statistical analysis was performed in IBM SPSS Statistics 20.

## Results

**Fig. 1:**
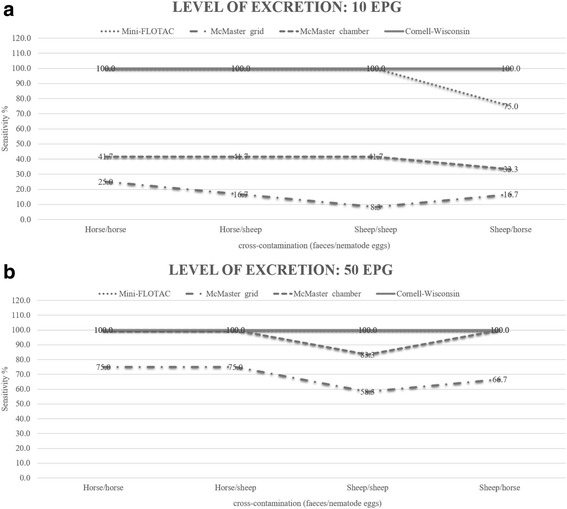
Analytical sensitivity (% of positive test results across the replicates) of each FEC technique using nematode egg suspensions of 10 EPG for the four cross-contaminations (**a**) and of 50 EPG for the four cross-contaminations (**b**)

The study involving 768 counts showed that at all egg concentrations the Mini-FLOTAC and Cornell-Wisconsin had 100% analytical sensitivity (using either sheep or horse faeces contaminated with nematode eggs). Instead, McMaster grid and chamber showed an analytical sensitivity of 100% only for concentrations greater than 200 EPG (the analytical sensitivity ranged from 8.3% to 75.0% at lowest concentration of eggs) (Fig. 1a, b). Spearman’s rank correlation showed a significant (*p* < 0.05) positive relationship between observed EPG values and true EPG values for all methods and for all types of cross-contamination, but the Rho values ranged from 0.91 for McMaster grid to 0.97 for Mini-FLOTAC. Additional files show mean of eggs (X), standard deviation (SD) and coefficient of variation (CV%) recovered by Mini-FLOTAC, McMaster and Cornell-Wisconsin for each EPG level and for each contamination [see Additional files 1, 2]. The mean of precision (CV%) and accuracy (X) for each method is presented in Tables 2 and 3.

**Table 2:** Mean CV% for Mini-FLOTAC, McMaster and Cornell-Wisconsin at the different egg count levels and for each method evaluated in this study

Method	10 EPG	50 EPG	200 EPG	500 EPG
Mini-FLOTAC	49.6%	10.9%	8.1%	3.1%
McMaster *grid*	248.6%	90.5%	39.9%	17.3%
McMaster* chamber*	135.6%	51.4%	23.1%	10.9%
Cornell-Wisconsin	33.4%	16.6%	51.8%	5.2%

**Table 3:** Mean number of detected eggs for Mini-FLOTAC, McMaster and Cornell-Wisconsin at the different egg count levels and for each method evaluated in this study

Method	10 EPG	50 EPG	200 EPG	500 EPG
Mini-FLOTAC	9	45	192	409
McMaster *grid*	8	49	179	492
McMaster *chamber*	7	39	167	461
Cornell-Wisconsin	4	19	104	248

**Fig. 2:**
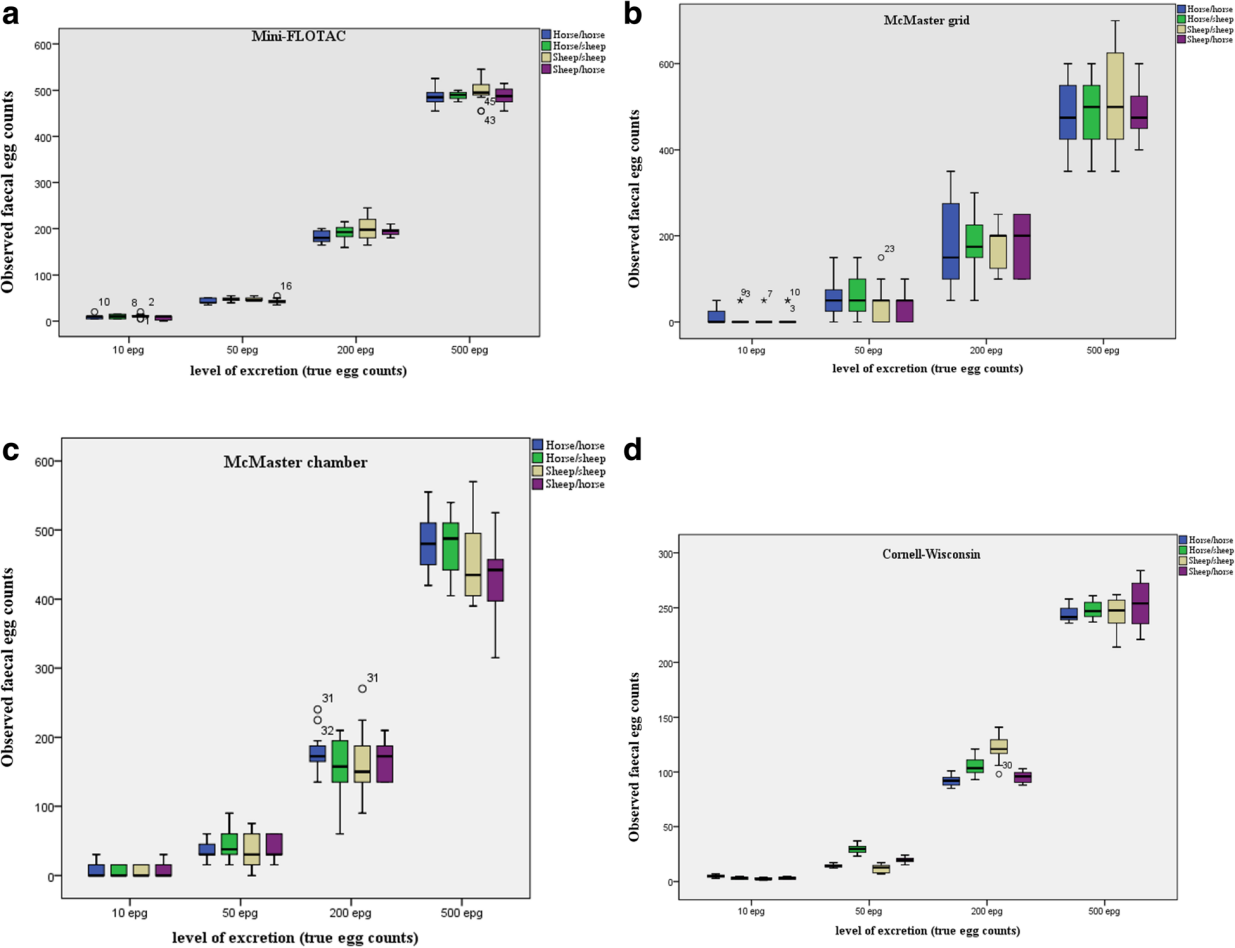
Boxplots of observed faecal egg counts (y axis) with: Mini-FLOTAC method (**a**), McMaster grid (**b**), McMaster chamber (**c**), Cornell-Wisconsin (**d**) for the four 4 levels of egg excretion (x-axis)

Fig. 2a-d show the boxplot of the observed EPG at each level of egg excretion for Mini-FLOTAC, McMaster grid, McMaster chamber and Cornell-Wisconsin, respectively. The length of boxplots of Mini-FLOTAC technique was very narrow for each contamination level and for all cross-contaminations showing a high precision and accuracy compared to the other techniques.

Sheep faeces had a mean (± standard deviation, SD) of 5.1 ± 0.14 g (maximum 5.1 g, minimum 4.8 g), while horse faeces had an average (± SD) of 5.0 ± 0.11 (maximum 5.2 g, minimum 4.9 g), thus demonstrating a good repeatability of the Fill-FLOTAC for weighing faecal samples..

At the lower level of eggs (10 EPG), CV% was high and exceeded 100% in McMaster grid and chamber methods. Furthermore, using McMaster grid and chamber methods were found negative results from the analysis of replicates, whereas the other methods never detected negative results.

## Discussion

Regarding the recovery of eggs, 100% of nematode eggs from sheep were recovered when added to egg-free sheep faeces, but only 91.0% were recovered from horse faeces. There was a significant difference between recovery of nematode eggs of sheep from sheep faeces and from horse faeces. When nematode eggs from horses were added to sheep faeces the recovery was 95.9%, but reduced egg counts (90.5%) were found when added to horse faeces. Noel et al. [15] performed a study on the percentage of recovery of eggs using Mini-FLOTAC technique for the diagnosis of equine strongyles and recovered 42.6% of the eggs. As discussed by Cringoli et al. [11], various factors might explain the difference between results presented in this study and results presented by Noel et al. [15]; in fact, one of the main limitations of Mini-FLOTAC technique, as with any copromicroscopic technique based on flotation (e.g. simple flotation, Wisconsin, and McMaster), is that the selection of fixative and duration of faecal preservation before Mini-FLOTAC analysis, the procedure of egg isolation and the choice of the flotation solution might influence the performance of the Mini-FLOTAC technique, specifically affecting the percentage of parasitic elements recovered [11]. The very poor performance of the Cornell-Wisconsin method indicates that this should not be used in future for counting equine nematode eggs, a conclusion also reached for bovine nematodes [4]. The McMaster technique is adequate if egg counts are greater than 50 EPG, but it is not satisfactory for lower counts which could be important if looking for the beginning AR. These results are similar to Vadlejch et al. [16] who compared the accuracy and precision of different McMaster methods for diagnosis of *Teladorsagia circumcincta* in sheep and confirmed that this method detected negative samples at lower concentrations. Under-estimation of FEC occurred when the entire McMaster chamber was examined rather than limited to the gridded area (Fig. 2b, c) whereas over-estimation of FEC occurred when the gridded area was examined, due to high multiplication factor. This is in agreement with Cringoli et al. [2] who observed aggregation of eggs to the center of McMaster slides, Morgan et al. [17] who described the Poisson distribution of nematode eggs in faecal suspensions and Kochanowsky et al. [14] that showed that the best limit of detection and analytical sensitivity and the lowest coefficients of variation were obtained with the use of the whole McMaster chamber variant. Only counting eggs in the gridded area appears to account for this aggregation at higher levels of egg densities; the number of eggs present at lower densities, however, was still underestimated. Finally CVs for McMaster grid and chambers were higher than other techniques for ovine and equine faeces, especially for lower counts, as yet reported by Noel et al. [15]. Also Dias de Castro et al. [18] and Scare et al. [19] showed that SD and CV values for significantly lower for Mini-FLOTAC than McMaster for detection of gastrointestinal nematode eggs in cattle and horses.

## Conclusions

In conclusion, Mini-FLOTAC combined with Fill-FLOTAC which provides an accurate method of weighing without need for a balance and filtering out debris, could be used for FEC on the farm as well as in the laboratory.

## Additional files

Additional file 1: Mean of eggs (X), Standard Deviation (SD), Coefficient of variation (CV%) recovered by Mini-FLOTAC, McMaster and Cornell-Wisconsin from horse faeces containing a predetermined number of nematode eggs extracted from horse and sheep faeces. (DOCX 14 kb)

Additional file 2: Mean of eggs (X), Standard Deviation (SD), Coefficient of variation (CV%) recovered by Mini-FLOTAC, McMaster and Cornell-Wisconsin from sheep faeces containing a predetermined number of nematode eggs extracted from horse and sheep faeces. (DOCX 14 kb)

## Abbreviations

EPG: Eggs per gram
FEC: Faecal egg counts
SD: Standard deviation
