# Supplementary material for: The recovery of added nematode eggs from horse and sheep faeces by three methods
Source: BMC Vet Res. 2018 Jan 5;14:7. doi: 10.1186/s12917-017-1326-7 (PMC5756441; doi:10.1186/s12917-017-1326-7)
Supplement: Supplementary file 1 — Mean of eggs (X), Standard Deviation (SD), Coefficient of variation (CV%) recovered by Mini-FLOTAC, McMaster and Cornell-Wisconsin from horse faeces containing a predetermined number of nematode eggs extracted from horse and sheep faeces. (DOCX 14 kb) [file 12917_2017_1326_MOESM1_ESM.docx]

**Additional file 1.**  Mean of eggs (X), Standard Deviation (SD), Coefficient of Variation (CV%) recovered by Mini-FLOTAC, McMaster and Cornell-Wisconsin from horse faeces containing a predetermined number of nematode eggs extracted from horse and sheep faeces.

| **Contamination of negative horse faeces** | Mini-FLOTAC | | | McMaster grid | | | McMaster chamber | | | Cornell-Wisconsin | | |
| --- | --- | --- | --- | --- | --- | --- | --- | --- | --- | --- | --- | --- |
|  | X | SD | CV(%) | X | SD | CV(%) | X | SD | CV(%) | X | SD | CV(%) |
| **10 EPG GIN from *horse*** | 8.75 | 4.33 | 49.49 | 12.50 | 22.61 | 180,88 | 7.50 | 10.11 | 134.80 | 5.08 | 1.16 | 22.91 |
| **10 EPG GIN from *sheep*** | 9.58 | 4.50 | 46.97 | 8.33 | 19.46 | 233,61 | 6.25 | 7.72 | 123.52 | 3.33 | 1.15 | 34.64 |
| **50 EPG GIN from *horse*** | 43.33 | 5.37 | 12.38 | 54.17 | 45.02 | 83.11 | 35.00 | 14.77 | 42.20 | 14.08 | 1.44 | 10.25 |
| **50 EPG GIN from *sheep*** | 47.08 | 4.50 | 9.56 | 58.33 | 46.87 | 80.35 | 45.00 | 22.16 | 49.24 | 29.42 | 3.90 | 13.24 |
| **200 EPG GIN from *horse*** | 182.92 | 13.05 | 7.13 | 179.17 | 101.04 | 56.30 | 178.75 | 29.63 | 16.58 | 92.17 | 4.86 | 5.27 |
| **200 EPG GIN from *sheep*** | 191.25 | 16.53 | 8.64 | 183.33 | 65.13 | 35.53 | 156.25 | 45.43 | 29.08 | 105.33 | 8.22 | 7.80 |
| **500 EPG GIN from *horse*** | 486.25 | 18.60 | 3.83 | 479.17 | 72.25 | 15.70 | 481.25 | 40.18 | 8.35 | 244.08 | 7.23 | 2.96 |
| **500 EPG GIN from *sheep*** | 488.75 | 7.72 | 1.58 | 479.17 | 81.07 | 16.92 | 477.50 | 42.83 | 8.97 | 248.42 | 7.83 | 3.15 |
