# Supplementary material for: The recovery of added nematode eggs from horse and sheep faeces by three methods
Source: BMC Vet Res. 2018 Jan 5;14:7. doi: 10.1186/s12917-017-1326-7 (PMC5756441; doi:10.1186/s12917-017-1326-7)
Supplement: Supplementary file 2 — Mean of eggs (X), Standard Deviation (SD), Coefficient of variation (CV%) recovered by Mini-FLOTAC, McMaster and Cornell-Wisconsin from sheep faeces containing a predetermined number of nematode eggs extracted from horse and sheep faeces. (DOCX 14 kb) [file 12917_2017_1326_MOESM2_ESM.docx]

**Additional file 2.**  Mean of eggs (X), Standard Deviation (SD), Coefficient of Variation (CV%) recovered by Mini-FLOTAC, McMaster and Cornell-Wisconsin from sheep faeces containing a predetermined number of nematode eggs extracted from horse and sheep faeces.

| **Contamination of negative sheep faeces** | Mini-FLOTAC | | | McMaster grid | | | McMaster chamber | | | Cornell-Wisconsin | | |
| --- | --- | --- | --- | --- | --- | --- | --- | --- | --- | --- | --- | --- |
|  | X | SD | CV(%) | X | SD | CV(%) | X | SD | CV(%) | X | SD | CV(%) |
| **10 EPG GIN from sheep** | 10.83 | 4.17 | 38.53 | 4.17 | 14.43 | 346.41 | 6.25 | 7.72 | 123,58 | 2.50 | 1.09 | 43.48 |
| **10 EPG GIN from horse** | 7.08 | 4.50 | 63.55 | 8.33 | 19.46 | 233.55 | 6.25 | 10.03 | 160,45 | 3.25 | 1.06 | 32.47 |
| **50 EPG GIN from *sheep*** | 47.92 | 3.96 | 8.27 | 41.67 | 46.87 | 112,49 | 36.25 | 26.72 | 73.72 | 11.58 | 3.48 | 30.01 |
| **50 EPG GIN from *horse*** | 42.92 | 5.82 | 13.57 | 41.67 | 35.89 | 86.13 | 40.00 | 16.10 | 40.24 | 19.50 | 2.54 | 13.03 |
| **200 EPG GIN from sheep** | 199.17 | 24.20 | 12.15 | 175.00 | 54.36 | 31.06 | 163.75 | 48.06 | 29.35 | 121.50 | 11.76 | 9.68 |
| **200 EPG GIN from horse** | 193.75 | 8.56 | 4.42 | 179.17 | 65.57 | 36.60 | 167.50 | 29.19 | 17.43 | 95.25 | 5.03 | 5.28 |
| **500 EPG GIN from sheep** | 497.92 | 26.84 | 5.39 | 520.83 | 117.66 | 22.59 | 453.75 | 62.38 | 13.75 | 244.92 | 15.06 | 6.15 |
| **500 EPG GIN from horse** | 488.33 | 18.75 | 3.84 | 487.50 | 67.84 | 13.92 | 432.50 | 54.59 | 12.62 | 253.25 | 21.46 | 8.47 |
